# Prevalence and risk factors for hepatitis E virus infection in blood donors: a nationwide survey in Italy, 2017 to 2019

**DOI:** 10.2807/1560-7917.ES.2022.27.22.2100516

**Published:** 2022-06-02

**Authors:** Enea Spada, Matteo Simeoni, Antonio Martina, Ilaria Pati, Umbertina Villano, Daniela Adriani, Agnese D’Angiò, Elena Tritarelli, Stefania Taffon, Stefania Bellino, Stefano Boros, Roberta Urciuoli, Francesca Masiello, Giuseppe Marano, Roberto Bruni, Patrizio Pezzotti, Anna Rita Ciccaglione, Simonetta Pupella, Vincenzo De Angelis, Giulio Pisani

**Affiliations:** 1 Department of Infectious Diseases, Istituto Superiore di Sanità, Rome, Italy; 2 National Centre for the Control and Evaluation of Medicines, Istituto Superiore di Sanità, Rome, Italy; 3 Italian National Blood Centre, Istituto Superiore di Sanità, Rome, Italy

**Keywords:** blood donor, HEV, Italy, prevalence, risk factor, zoonosis

## Abstract

**Background:**

In high-income countries, hepatitis E virus (HEV) infection is mainly a zoonosis. However, it is also transfusion-transmissible and some countries, but not Italy, have introduced HEV screening for blood donations.

**Aim:**

We assessed HEV infection prevalence and risk factors in a nationwide sample of Italian blood donors.

**Methods:**

We selected 107 blood establishments (BE) distributed in the 20 Italian regions by a stratified two-stage design and invited them to participate in the study. Donors were tested for anti-HEV IgG and IgM and HEV RNA. Sociodemographic data and risk factors were collected through a questionnaire.

**Results:**

Overall, 60 BE from 60 provinces in 19 Italian regions joined the study. We assessed HEV markers in 7,172 blood donors, of whom 6,235 completed the questionnaire. Overall crude and adjusted anti-HEV IgG prevalences were 8.3% and 5.5%, respectively. Overall anti-HEV IgM prevalence was 0.5%, while no blood donor was HEV RNA-positive. Anti-HEV IgG prevalence varied widely among regions (range: 1.3%–27.20%) and hyperendemic prevalences (> 40%) were detected in some provinces in two regions. Older age (AOR = 1.81; 95% CI: 1.36–2.41), foreign nationality (AOR = 2.77; 95% CI: 1.06–7.24), eating raw pork liver sausages (AOR = 2.23; 95% CI: 1.55–3.20) and raw homemade sausages (AOR = 3.63; 95% CI: 2.50–5.24) were independent infection predictors.

**Conclusion:**

Italian blood donors showed a low to moderate HEV seroprevalence. High levels in some regions and/or provinces were mainly attributable to eating habits. Prevention should include avoiding consumption of raw or undercooked meat and safe production of commercial pork products.

## Introduction

Hepatitis E virus (HEV) is a small, non-enveloped single-stranded RNA virus belonging to the *Hepeviridae* family, *Orthohepevirus* genus and *Orthohepevirus A* species. There are eight distinct *Orthohepevirus A* genotypes of which five (HEV-1–4 and -7) can infect humans. HEV-1 and -2 infect only humans. The other three genotypes also infect other animals such as pigs, wild boars, deer, rabbits (HEV-3 and -4) and camelids (HEV-7) [1,2].

HEV-1 and -2 are prevalent in low- and lower-middle-income areas where transmission is mainly faecal-oral, usually via contaminated water, often causing epidemics. Overt disease usually affects young adults and can be severe in pregnant and patients with liver disease [3-5]. HEV-3 is spread worldwide, whereas HEV-4 is prevalent in Asia but is also present in Europe. Usually, HEV-3 and -4 are transmitted by food through ingestion of raw or undercooked meat and organs (especially liver and offal) of infected host animals (mostly pig, wild boar, deer and rabbit), or by direct contact with infected animals, affecting workers on pig farms or in slaughterhouses, and hunters. Food-borne transmission can also occur by consuming faecally contaminated vegetables, fruits, molluscs and drinking water. Finally, inter-human transmission of HEV-3 and HEV-4 by transfusion of blood or blood products and via solid organ transplantation has been demonstrated [3-5]. HEV-7 infection was first detected in a person consuming camel milk and meat [2]. Most HEV-3 or HEV-4 infections are asymptomatic; clinical disease mainly affects people older than 40 years. In immunocompromised patients, these infections (in most cases HEV-3 and sometimes HEV-4 and HEV-7) can become chronic, even leading to cirrhosis [2-5].

Studies in the general population and among blood donors in different countries have shown heterogeneous (from < 5% to > 50%) prevalence levels of anti-HEV IgG (indicating past infection), with wide differences even in the same country [5-7]. This variability was attributed to the studies' geographical location, the included population and mainly the performance characteristics of the anti-HEV IgG assay used [4-7]. The Wantai anti-HEV IgG is the most commonly used assay wordwide and has high specificity and sensitivity, whereas non-Wantai assays tend to underestimate HEV seroprevalence [4-7]. A recent metanalysis of studies using the Wantai anti-HEV IgG assay to analyse the general population and blood donors in 15 high-income countries found prevalences ranging from 4.2 to 52.5% [7]. In Italy, an earlier nationwide blood donor survey using this assay found an overall prevalence of 8.7%, with an interregional variation from 2.2% to 22.8% [8].

The risk of transfusion-transmitted HEV infection has led to a large amount of prevalence studies on blood donors and donations almost everywhere in the world. As a result of these studies, eight European countries have since 2012 implemented universal or selective (i.e. on blood intended for transfusion to immunocompromised patients) HEV RNA screening of blood donations [9]. In Italy, routine HEV blood donation screening has not been introduced so far.

This study aimed to estimate the prevalence of HEV infection in a nationwide sample of Italian blood donors and to identify risk factors associated with anti-HEV IgG seropositivity.

## Methods

### Study characteristics

The study was the result of a collaboration among centres and departments of the Istituto Superiore di Sanità, including the National Centre for the Control and Evaluation of Medicines, the Department of Infectious Disease and the National Blood Centre, that got the support and cooperation of Italian blood establishments (BE).

### Participants and study design

To guarantee maximum territorial representation, both at regional and provincial level, a sample of 107 BE distributed throughout the country (one per Italian province) were selected and invited by the National Blood Centre to participate in the study. We used a stratified (by province) two-stage (BE and then donors) design. In provinces that had more than one BE, the choice was based on a probability selection proportional to the 2015 volume of each BE. Overall, 60 BE from 60 provinces joined the study.

Participants of each BE were not randomly selected; usually they were enrolled on 1 day, although BE were free to adapt the process to their needs. We did not use stratification by age and sex.

Enrolment of blood donors continued from April 2017 to March 2019. All donors who agreed to participate were asked to complete a questionnaire collecting socio-demographic information and data on risk factors.

### Virological assays

All plasma samples were tested for anti-HEV IgG antibodies with the Wantai HEV-IgG ELISA (Wantai, Biologic Pharmacy Enterprise, Beijing, China). To save money and personnel resources, anti-HEV IgM antibodies were assessed by Wantai HEV-IgM ELISA in all samples in provinces with an anti-HEV IgG prevalence ≥ 15%; while in those with a prevalence < 15%, only IgG-positive samples were tested. Both the IgG and IgM anti-HEV assays target recombinant antigens expressed from the ORF2 region.

All plasma samples (0.5 mL) were tested for HEV RNA with the Procleix HEV assay kit on fully automated Procleix Panther system instrumentation (Hologic, Inc., San Diego, United States (US)/Grifols Diagnostic Solutions, Inc., Emeryville, US). The analytical method has a 95% limit of detection (95% LOD) of 7.9 IU/mL and can detect all four HEV genotypes with a 95% LOD in samples with a HEV RNA concentration between 7.9 and 17.7 IU/mL.

Serum samples from all donors participating in the study were sent by their respective BEs to the Institute Superiore di Sanità where all virological analyses were carried out.

### Statistical analysis

We calculated prevalences of anti-HEV IgG and their 95% confidence intervals (CI), both overall and for each Italian region, using a logistic regression model including BE as random effect. Risk factors for HEV infection were evaluated using both univariable and multivariable mixed-effects logistic regression models with BE as random effect, according to a two-stage design.

Statistical analysis was carried out using Stata version 16 (Stata Corporation, College Station, Texas, US).

## Results

A total of 7,172 blood donors (70% male, 30% female; median age: 43 years; age range: 18-68) were enrolled in the study and assessed for HEV infection markers; of these, 6,235 completed the questionnaire. Participant blood donors from different regions were comparable in term of age and sex.

### Seroprevalence of anti-HEV IgG 

Table 1 shows overall and regional results as both crude prevalences (proportion of positive blood donors over the total number of tested) and adjusted prevalences (calculated by logistic regression model including BE as random effect). The overall crude and adjusted prevalences were 8.3% (597/7,172) and 5.5%, respectively. The adjusted anti-HEV IgG prevalence was comparable between male and female donors (Table 1). Past infection prevalence increased significantly with age, ranging from 3.2% among donors aged 18–34 years to 7.8 among donors 55 years and older (Table 2).

**Table 1:** Prevalence of HEV infection markers in blood donors, by region of residence, Italy, 2017–2019 (n = 7,172)

Regions^a^	Number of donors			Anti-HEV IgG			Crude anti-HEV IgG prevalence (%)			Adjusted anti-HEV IgG prevalence^b^ (%)						Anti-HEV IgM			HEV RNA
	Total	M	F	Total	M	F	Total	M	F	Total	95% CI	M	95% CI	F	95% CI	n	%	Also IgG-positive	n
Piedmont^c^	443	318	124	40	33	7	9.0	10.4	5.6	9.0	6.7–12.1	10.3	7.4–14.2	5.6	2.7–11.4	1	0.2	1	0
Aosta Valley	60	38	22	2	2	0	3.3	5.3	0.0	3.3	0.8–12.4	5.3	1.3–18.7	0	0.0–0.0	0	0	0	0
Lombardy	421	264	157	26	16	10	6.2	6.1	6.4	6.2	4.2–8.9	6.1	3.7–9.7	6.4	3.5–11.4	1	0.2	1	0
Trentino-Alto Adige	356	279	77	8	6	2	2.2	2.2	2.6	2.2	1.1–4.4	2.2	1.0–4.7	2.6	0.7–9.8	0	0	0	0
Veneto	482	273	209	15	9	6	3.1	3.3	2.9	3.1	1.9–5.1	3.3	1.7–6.2	2.9	1.3–6.2	1	0.2	1	0
Liguria	86	58	28	4	3	1	4.7	5.2	3.6	4.7	1.8–11.7	5.2	1.7–14.8	3.6	0.5–21.4	0	0	0	0
Emilia Romagna^c^	499	294	204	18	13	5	3.6	4.4	2.5	3.4	1.7–6.5	4.2	2.1–8.3	2.5	1.0–5.8	0	0	0	0
Tuscany^d^	758	488	266	63	42	21	8.3	8.6	7.9	7.6	5.0–11.4	7.9	4.9–12.4	7.9	5.0–12.3	0	0	0	0
Umbria	153	89	64	6	5	1	3.9	5.6	1.6	3.9	1.8–8.5	5.6	2.4–12.8	1.6	0.2–10.3	1	0.6	1	0
Marche^c^	641	456	184	97	72	25	15.1	15.8	13.6	11.9	6.6–20.5	11.2	5.5–21.5	13.6	9.3–19.3	13	2	9	0
Lazio	303	226	77	27	19	8	8.9	8.4	10.4	7.0	2.0–21.6	8.4	5.4–12.8	10.4	5.3–19.4	1	0.3	1	0
Abruzzo	584	376	208	175	134	41	30.0	35.6	19.7	27.2	18.0–38.9	31.9	22.7–42.7	20.3	13.5–29.3	10	1.7	7	0
Molise^e^	112	86	24	13	11	2	11.6	12.8	8.3	11.6	6.9–19.0	12.5	7.1–21.2	8.3	2.1–27.9	0	0	0	0
Campania^c^	598	440	157	15	11	4	2.5	2.5	2.5	2.5	1.5–4.1	2.2	0.9–5.5	2.5	1.0–6.6	0	0	0	0
Apulia^c^	497	384	112	12	10	1	2.4	2.6	0.9	2.4	1.4–4.2	2.9	1.6–5.1	0.6	0.0–20.7	0	0	0	0
Basilicata	56	35	21	3	1	2	5.4	2.9	9.5	5.3	1.7–15.3	2.9	0.4–17.7	9.5	2.4–31.1	0	0	0	0
Calabria	150	129	21	2	2	0	1.3	1.6	0.0	1.3	0.3–5.2	1.6	0.4–6.0	0	0.0–0.0	0	0	0	0
Sicily^c^	526	408	117	13	8	5	2.5	2.0	4.3	2.5	1.4–4.2	2.0	1.0–3.9	4.3	1.8–9.9	1	0.2	1	0
Sardinia^f^	447	336	106	58	49	9	13.0	14.6	8.5	13.3	6.7–24.6	14.3	7.0–26.9	8.5	4.5–15.5	4	0.9	4	0
**Total^g,h^**	**7,172**	**4,977**	**2,178**	**597**	**446**	**150**	**8.3**	**9.0**	**6.9**	**5.5**	**4.2–7.1**	**5.6**	**4.2–7.5**	**5.5**	**4.1–7.3**	**33**	**0.5**	**26**	**0**

CI: confidence interval; F: female; HEV: hepatitis E virus; M: male.

^a^ Regions are listed in the order north to south, ending with the island regions.

^b^ Prevalence of anti-HEV IgG and 95% confidence intervals, both overall and for each Italian region, were calculated using the logistic regression model including blood establishment as random effect.

^c^ Gender data were not available for one blood donor who tested anti-HEV IgG-negative.

^d^ Sex data were not available for four blood donors who all tested anti-HEV IgG-negative.

^e^ Sex data were not available for two blood donors who both tested anti-HEV IgG-negative.

^f^ Sex data were not available for five blood donors who all tested anti-HEV IgG-negative.

^g^ Sex data were not available for 17 blood donors who all tested anti-HEV IgG-negative

^h^ Percentage of anti-HEV IgM-positive donors is calculated as ratio between number of IgM-positive donors and total donors.

**Table 2:** Anti-HEV IgG prevalence among blood donors, by age group, Italy, 2017–2019 (n = 7,064)

Age group (years)	Blood donors^a^ (n)	Anti-HEV IgG (n)	Anti-HEV IgG^b^ (%)	95% CI^b^
18–34	1,984	94	3.2	2.1–4.9
35–44	1,821	122	4.2	2.8–6.2
45–54	2,157	228	6.6	4.8–9.1
≥ 55	1,102	146	7.8	5.3–11.3

CI: confidence interval; HEV: hepatitis E virus.

^a^ Age data were not available for 108 blood donors.

^b^ Prevalence of anti-HEV IgG and 95% CI were calculated using the logistic regression model including blood establishment as random effect.

**Figure 1:**
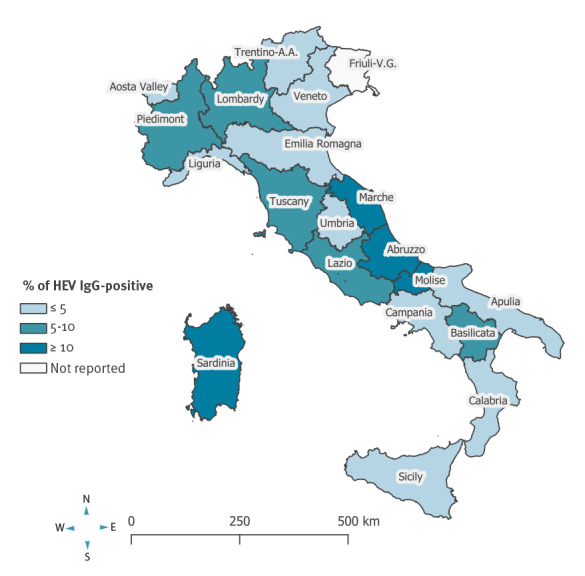
Prevalence of anti-HEV IgG in blood donors, by region of residence, Italy, 2017–2019 (n = 7,172)

We found considerable interregional variation in the prevalence of past HEV infection. Adjusted anti-HEV IgG seroprevalence ranged from 27% in Abruzzo to 1.3% in Calabria. Besides Abruzzo, the seroprevalence exceeded 10% in Sardinia, Marche and Molise. A prevalence between 5% and 10% were found in Basilicata, Lombardy, Lazio, Tuscany and Piedmont. In all remaining regions, the prevalence was lower than 5% (Figure 1 and Table 1).

HEV: hepatitis E virus.

**Figure 2:**
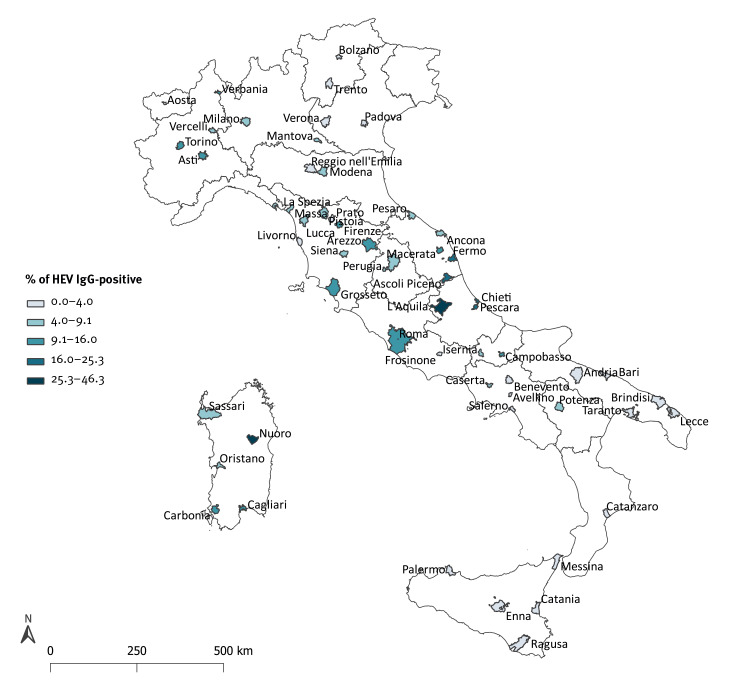
Prevalence of anti-HEV IgG in blood donors, by province of blood establishment, Italy, 2017–2019 (n = 7,172)

Figure 2 shows the anti-HEV IgG prevalence by province of BE location. There was some intraregional variability in prevalence in several regions (e.g. Tuscany, Sardinia, Piedmont and Marche). Of note, some provinces showed an uncommonly high prevalence, for example Nuoro (19/43; 44.2%; 95% CI: 29.1–60.1) and L’Aquila (112/279; 40.1%; 95% CI: 34.3–46.1), located in Sardinia and Abruzzo, respectively. The prevalence in several provinces in Central Italy was two- to three times higher than the overall national prevalence level: Pescara (25/102; 24.5%; 95% CI: 16.5–34.0) and Chieti (38/203; 18.7%; 95% CI: 13.6–24.8) in Abruzzo and Ascoli Piceno (50/200; 25.0%; 95% CI: 19.2–31.6) and Fermo (14/75; 18.7%; 95% CI: 10.6–29.3) in Marche.

### Demographic and risk factor variables associated with anti-HEV IgG positivity

We applied univariable and multivariable mixed-effects logistic regression models to evaluate sociodemographic variables and recognised risk factors associated with anti-HEV IgG positivity. Almost all characteristics considered resulted, at univariate level, as significantly associated (p < 0.05) with past HEV infection (Table 3). In the multivariable model, adjusting for all covariates and including BE as random effect, the risk of past HEV infection was higher in people 40 years and older (adjusted odds ratio (AOR) = 1.81; 95% confidence interval (CI): 1.36–2.41), those with foreign nationality (AOR = 2.77; 95% CI: 1.06–7.24) and in people eating uncooked pork liver sausages (AOR = 2.23; 95% CI: 1.55–3.20) and homemade pork sausages (AOR = 3.63; 95% CI: 2.50–5.24).

**Table 3:** Univariable and multivariable mixed-effects logistic regression model analysing socio-demographic characteristics and risk factors^a^ associated with HEV infection in blood donors, Italy 2017–2019 (n = 6,235)

		n	IgG anti-HEV (%)	Univariable logistic model			Multivariable logistic model		
				OR	95% CI	p value	OR	95% CI	p value
Sex	Female	1,870	7.3	1	Reference		1	Reference	
	Male	4,365	8.9	1.25	1.01–1.56	0.041	1.15	0.85–1.56	0.364
Age (years)	< 40	2,421	5.4	1	Reference		1	Reference	
	≥ 40	3,774	10.3	1.94	1.56–2.41	< 0.0001	1.81	1.36–2.41	< 0.001
Nationality	Italian	5,883	8.6	1	Reference		1	Reference	
	Foreign	74	14.9	1.81	0.91–3.60	0.089	2.77	1.06–7.24	0.038
District of birth	Urban area	5,065	7.9	1	Reference		1	Reference	
	Rural area	1,170	10.6	1.33	1.05–1.67	0.017	0.87	0.60–1.27	0.479
District of residence	Urban area	4,848	8.0	1	Reference		1	Reference	
	Rural area	1,387	10.0	1.25	1.00–1.57	0.049	1.13	0.79–1.60	0.506
Work experience with wild or farm animals	No	5,350	7.9	1	Reference		1	Reference	
	Yes	669	13.5	1.58	1.22–2.05	0.001	1.02	0.69–1.51	0.913
Domestic contact with animals^b^	No	4,187	7.6	1	Reference		1	Reference	
	Yes	1,663	9.1	1.14	0.92–1.42	0.221	0.79	0.56–1.11	0.171
Contacts with pigs, wild boars or wild animals	No	4,948	6.9	1	Reference		1	Reference	
	Yes	1,051	13.1	1.55	1.23–1.94	< 0.001	0.99	0.67–1.46	0.955
Contacts with other animals^c^	No	3,974	6.7	1	Reference		1	Reference	
	Yes	2,063	10.6	1.45	1.19–1.77	< 0.001	0.98	0.68–1.42	0.923
Hunting	No	5,898	8.0	1	Reference		1	Reference	
	Yes	270	16.7	1.99	1.39–2.86	< 0.001	1.61	0.99–2.62	0.056
Gardening	No	3,753	6.8	1	Reference		1	Reference	
	Yes	2,213	11.2	1.55	1.28–1.89	< 0.001	1.23	0.88–1.71	0.222
Vegetable gardening	No	4,229	7.0	1	Reference		1	Reference	
	Yes	1,942	11.2	1.45	1.19–1.76	< 0.001	0.90	0.61–1.33	0.588
Eating pork liver salami^d^	No	4,983	6.1	1	Reference		1	Reference	
	Yes	733	21.7	2.95	2.31–3.77	< 0.001	1.25	0.86–1.82	0.238
Eating uncooked liver sausages^d^	No	5,098	5.5	1	Reference		1	Reference	
	Yes	919	24.2	3.67	2.92–4.62	< 0.001	2.23	1.55–3.20	< 0.001
Eating uncooked pork sausages^d^	No	2,418	3.1	1	Reference		1	Reference	
	Yes	3,668	12.0	3.51	2.69–4.58	< 0.001	1.22	0.81–1.84	0.343
Eating uncooked wild boar sausages^d^	No	4,317	6.6	1	Reference		1	Reference	
	Yes	1,697	13.3	1.80	1.47–2.21	< 0.001	0.89	0.67–1.20	0.449
Eating homemade sausages^d^	No	2,613	3.3	1	Reference		1	Reference	
	Yes	2,560	13.6	4.15	3.20–5.39	< 0.001	3.63	2.50–5.27	< 0.001
Eating uncooked game meat^d,e^	No	4,779	7.4	1	Reference		1	Reference	
	Yes	1,333	11.9	1.40	1.14–1.73	0.002	1.06	0.79–1.42	0.691
Eating raw seafood	No	3,566	7.7	1	Reference		1	Reference	
	Yes	2,293	9.9	1.19	0.98–1.46	0.078	0.93	0.71–1.22	0.607
Eating vegetables from your own garden	No	3,241	6.9	1	Reference		1	Reference	
	Yes	2,549	10.6	1.38	1.13–1.69	0.001	1.05	0.73–1.51	0.798
Travel abroad	No	1,661	8.4	1	Reference		1	Reference	
	Yes	4,476	8.5	0.94	0.75–1.18	0.603	0.97	0.70–1.33	0.829

AOR: adjusted odds ratio; CI: confidence interval; HEV: hepatitis E virus; OR: odds ratio.

Missing values for each variable were not included in the analysis, they ranged from 0.6% to 8.3% for the evaluated risk factors.

^a^ Exposure to risk factors was assessed over a lifetime.

^b^ I.e. presence of an animal in the family, including farms, family livestock, etc.

^c^ Horses, sheep, cattle, goats, cats, dogs etc.

^d^ Raw or undercooked.

^e^ Wild boar, deer etc.

The anti-HEV IgG prevalence in survey participants with nationalities other than Italian was almost twofold higher than in Italian donors; the highest prevalences (20%) were detected among blood donors from central-eastern and north-western Europe (Supplementary Table S1).

### Seroprevalence of anti-HEV IgM 

Overall, 33 of 7,172 (0.5%) donors were anti-HEV IgM-positive; of them, 26 were also anti-HEV IgG-positive. Taking into account the anti-HEV IgM testing strategy adopted in this study, overall 1,816 blood donors were tested for this marker. Thus the seroprevalence in those actually tested for anti-HEV IgM was 1.8% (33/1,816; 95% CI: 1.30–2.54). Regional anti-HEV IgM prevalence ranged from 0 to 2%; the highest proportions were found in Marche, Abruzzo and Sardinia (Table 1). The seven blood donors who tested anti-HEV IgM- but not IgG-positive were found in Abruzzo and Marche (Table 1).

### Prevalence of HEV RNA 

No blood donors were positive for HEV RNA.

### Prevalence of any HEV markers

Considering the above reported HEV RNA result and the seven participants positive only for anti-HEV IgM, the overall crude prevalence for any HEV infection marker was 8.4% (604/7,172; 95% CI: 7.80–9.9).

## Discussion

This study was the second nationwide survey assessing HEV infection prevalence among Italian blood donors; a similar study was conducted during 2015 and 2016 [8]. However, unlike that earlier study, this survey also aimed to identify risk factors for infection.

In this study, the overall crude and adjusted anti-HEV IgG prevalences among Italian blood donors were 8.3% and 5.5%, respectively. We observed considerable interregional variability in the anti-HEV IgG prevalence, along with wide intraregional variability in some regions. The overall prevalence of anti-HEV IgM and HEV RNA was 0.5% and 0%, respectively.

Comparing our prevalence data with those from previous studies performed in Italy and abroad is difficult because of the difference in study population (e.g. general population, blood donors etc.) and anti-HEV IgG assays employed in the various studies. Instead, it seems correct to compare our data with those of similar studies conducted in blood donors where the anti-HEV IgG Wantai assay was used.

Only three previous Italian studies have these features [8,10,11]. The first was the above-mentioned nationwide survey [8], which detected very similar overall crude anti-HEV IgG and IgM prevalence levels. Most of the regional anti-HEV IgG prevalences were also comparable between the two nationwide surveys, albeit with significant variations for several regions. This was probably due to two reasons: the different selection and number of provincial BE participating for each region and the occurrence of temporal prevalence variations (see below). Undoubtedly, in two regions (Calabria and Umbria), the low participation of blood donors may have affected the prevalence variation. The second study among blood donors employing the Wantai assay was performed in Sondrio (Lombardy), and it found a HEV IgG prevalence of 9.8% [10]. Unfortunately, the province of Sondrio was not selected to participate in our survey, thus hampering a direct data comparison. The third such Italian study was conducted during 2014 in L’Aquila (Abruzzo); the detected anti-HEV IgG prevalence was 49% [11]. This proportion is significantly higher than those found in the same town both in the present (40.1%) and in the earlier nationwide survey (31.6%) [8]. Temporal variations in anti-HEV IgG prevalence among blood donors in the same geographical area have already been reported in other countries, even across a longer time span [12,13]. Indeed, year-by-year and even seasonal variations in anti-HEV prevalence in the same geographical area may occur. For example, in this survey, samples from blood donors in L’Aquila were collected in three distinct periods: December 2017 to January 2018, September to October 2018, and February to March 2019. Anti-HEV IgG prevalences in these periods were 36% (28/77; 95% CI: 25.7–48.1), 48% (49/102; 95% CI: 8.0–58.1) and 34% (20/58; 95% CI: 22.5–48.1), respectively. The most rational explanation for such short-term temporal variations in prevalence is that donors of each group may have had a different history of HEV risk exposures over time, both in terms of quality (e.g. habit of eating pork liver sausages) and maybe quantity (e.g. opportunity for re-infections). However, we found no statistically significant differences between these three blood donor groups with respect to age, sex or other risk exposures. This was probably because number of specific exposures in these groups was not sufficient to allow detecting such differences.

In Europe, based on studies published during the 2010s, the highest anti-HEV IgG prevalence levels were found in France (22.4%; with some regional prevalences > 50%), Poland (43.5%), the Netherlands (27% and 31%) and Switzerland (20.4%) [12,14-18]. Prevalences between 10 and 20% were reported in Austria, Denmark, Norway, Spain and the United Kingdom (England and North Wales) [19-24]. Ireland and Scotland showed proportions lower than 10% [25,26]. In some of the above countries (e.g. France, Italy and Spain), anti-HEV IgG prevalence was assessed in donors or in the general population also using assays other than Wantai, and fairly similar prevalence figures were found [7]. Unfortunately, no seroprevalence survey in blood donors in Germany have to date been carried out using the Wantai assay [7], thus preventing a direct comparison with our data.

Thus, in this study we observed overall a crude and adjusted anti-HEV IgG prevalence of 8.3% and 5.5%, respectively. Nevertheless, areas with an uncommonly high prevalence exist also in Italy, resembling observations in France [14,27,28]. Despite the lack of a concerted satisfying definition of the levels of HEV endemicity, some areas in Europe with seroprevalences around 50% or more have been classified as hyperendemic [7,8,11,12,14,27]. Based on findings available to date, there are almost certainly two HEV-hyperendemic areas in Italy: one in Sardinia, the other in Abruzzo [8,11]. The earlier nationwide survey found anti-HEV IgG prevalences of 38.5% in Nuoro and 30.2% in Ozieri, both located in the central-eastern part of Sardinia [8]. In the present survey, anti-HEV IgG prevalence in Nuoro was 44.2%. Among blood donors in Abruzzo, especially in L’Aquila province, HEV prevalence levels have constantly been high since 2014 when the first HEV survey among blood donors was performed in this town [8,11]. Moreover, in a prospective study on blood donors from L’Aquila, the HEV infection incidence was 2.1 per 100 person-years, a prevalence two˗ to 10-fold higher than that found in other European countries in the general population and blood donors [29].

In this nationwide survey, as in the first one [8], we did not observe a high prevalence in areas with intensive pig breeding (i.e. Lombardy, Piedmont, Emilia-Romagna and Veneto), thus supporting the reported lack of correlation between living in areas with high-density pig farming and frequency of HEV in humans [8,12,16,23].

Although no participant tested HEV RNA-positive, 0.5% of them were positive for anti-HEV IgM, a key marker of acute or recent HEV infection [3,4,7]. As expected, the highest anti-HEV IgM prevalence levels, as well as the highest proportions of individuals positive only for IgM, were found in regions with the highest IgG prevalences (Abruzzo and Marche). This was due in part to the IgM testing strategy adopted in our study and in part to the increased likelihood of detecting people positive for IgM, including IgM alone, (i.e. with recent/acute infections) when performing cross-sectional studies in high-prevalence areas [12,30].

In our study, after adjustment for all covariates, older age was significantly associated with anti-HEV IgG positivity, which is in agreement with other data [6,12,16,18,23,25]. This was probably due to a lifetime cumulative HEV exposure. Foreign nationality was also independently associated with past HEV infection. The highest anti-HEV IgG prevalences (20%) were detected among migrants from both north-western and central-eastern Europe, who represented the most numerous groups of foreign people residing in Italy in the study period. Like others [12,14,28,31-33], we also observed that anti-HEV IgG positivity was associated with eating pork liver sausages and homemade sausages. The risk associated with eating pork sausages containing liver tissue is due to the liver being the main place of HEV replication and concentration. In Italy, certain eating habits have undoubtedly been favoured HEV spread in Abruzzo [11,29]. Also in Molise and Marche, pork and wild boar meat are part of the regional cuisine. In Marche, a high annual incidence of acute hepatitis E has been documented in recent years. Besides, an outbreak caused by consumption of undercooked pork sausages was reported during 2013 and 2014 [34]. The risk associated with eating homemade sausage may be related to the fact that these artisanal products may more frequently than industrial products contain pig liver, blood, offal and hog casings which can also be infected. Moreover, faeces or bile of infected animals may more easily contaminate pork meat, equipment and utensils during homemade processing [35]. Lastly, like others [36,37], we found an association between hunting and anti-HEV IgG positivity, but it was not statistically significant (p = 0.056). If skinning and disembowelling of infected animals such as wild boars or deer without gloves, hunters may have direct contact with body fluids or faeces [36,37]. In Italy, the population of wild boars has doubled in the last decade [38]. This increase has raised concerns about the impact on crops, animal and human health in many Italian regions, leading to the resumption of wild boar hunting.

In this context, the situation in Sardinia deserves attention. In both nationwide surveys, the highest prevalences were constantly detected in provinces located in the central-eastern part of this island [8], where areas with free-range pig farming are concentrated. Free-range farming allows frequent interactions between wild boars and pigs, resulting in possible virus transmission (e.g. HEV or African swine fever virus) and generation and circulation of feral pig-wild boar hybrids [39,40]. Pig-wild boar hybrids have social attitudes more similar to domestic pigs (diurnal), breed more regularly and have larger litters than pure wild boars, and therefore reach high population density. An uncontrolled increase in the wild boar population, free-range pig farming and wild boar-pig hybridisation can favour HEV transmission to domestic and wild animals and to humans and might ultimately led to a widespread environmental contamination with HEV [39,40]. However, we did not collect and analyse any data that would allow us to measure such a contamination. Therefore, the role of environmental contamination in the spread of HEV in high-endemic areas remains an interesting working hypothesis to be verified through further ad hoc studies.

According to French studies, also other unsuspected factors such as tap water might have a role in HEV spread, especially in high-prevalence areas [12,27]. In Italy, HEV has been found in urban wastewater, in river waters receiving wastewater discharges and in marine waters and shellfish [41,42]. In Europe, HEV has been detected in irrigation water from farms producing berries and leafy vegetables [43,44].

One of the main strengths of this study is that it was conducted on a large representative nationwide sample of blood donors and combined the investigation of HEV infection markers and recognised risk factors for infection [12,17]. To the best of our knowledge it is the only such study in which all markers of HEV infection (including IgM and HEV RNA) have been assayed.

Limitations were: One small region (2% of the national population) did not participate because its only BE did not join the study for logistical reasons. The number of participants in some regions was small, which may have influenced the estimated prevalence in these regions but not the overall national one. The questionnaire had not previously been tested in a subgroup of blood donors, some variables of the questionnaire could not be independent, and the evaluation of single food items could be affected by other ones (e.g. raw liver sausage could be correlated with eating similar foods). A total of 937 of the 7,172 blood donors (13%) did not complete the questionnaire. Missing data for the evaluated risk factors ranged from 0.6% to 8.3%. Finally, recall bias may have played a role in data collection.

## Conclusion

Our study confirmed a low to moderate anti-HEV IgG seroprevalence among Italian blood donors. However, important regional variations in prevalence and at least two well delimited hyperendemic areas also exist. Moreover, were detected a non-negligible frequency of acute or recent infections in high-prevalence regions. In this scenario, the adoption of prevention exposure measures at individual level (e.g. avoiding consumption of raw or undercooked pork meat and safe hunting activity), particularly for immunocompromised persons, and of safe procedures in the processing and production of pork meat products may be crucial in reducing the spread and clinical impact of HEV infection. Considering the study results, particularly the absolute lack of donors positive for HEV RNA (as in the first nationwide survey), the introduction of universal HEV RNA blood donation screening in Italy does not appear justified. Furthermore, pathogen reduction technologies effective on HEV should be applied if it has been the normal practice in the BE.

## Supplementary Data

146000 Supplement
