# Supplementary material for: Prevalence and risk factors for hepatitis E virus infection in blood donors: a nationwide survey in Italy, 2017 to 2019
Source: Euro Surveill. 2022 Jun 2;27(22):2100516. doi: 10.2807/1560-7917.ES.2022.27.22.2100516 (PMC9164674; doi:10.2807/1560-7917.ES.2022.27.22.2100516)
Supplement: Supplement [file 21-00516_SPADA_SupplementaryTable.pdf]

Eurosurveillance as supporting information alongside the article [Prevalence and risk factors for hepatitis E virus infection in blood donors: a nationwide survey in Italy, 2017 to 2019] on behalf of the authors who remain responsible for the accuracy and appropriateness of the content. The same standards for ethics, copyright, attributions and permissions as for the article apply. Supplements are not edited by *Eurosurveillance* and the journal is not responsible for the maintenance of any links or email addresses provided therein.

Supplementary Table 1. Anti-HEV IgG prevalence among foreign blood donors by place of birth. 2017-2019.

Place of birth	N.	N. IgG+	% IgG+
Central/East Europe	40	8	20.0
North/West Europe	10	2	20.0
Central/South America	14	1	7.1
Africa	5	0	0
Asia	3	0	0
ND	2	0	0

N., number of donors tested; N. IgG+, number of anti-HEV IgG positive donors; % IgG+, percentage of anti-HEV IgGpositive donors; ND, not determined.
